# How much is that doodle in the window? Exploring motivations and behaviours of UK owners acquiring designer crossbreed dogs (2019-2020)

**DOI:** 10.1186/s40575-022-00120-x

**Published:** 2022-05-24

**Authors:** E. Burnett, C. L. Brand, D. G. O’Neill, C. L. Pegram, Z. Belshaw, K. B. Stevens, R. M. A. Packer

**Affiliations:** 1 Department of Clinical Sciences and Services, The Royal Veterinary College, Hawkshead Lane, North Mymms, Hatfield, Herts AL9 7TA UK; 2 School of Veterinary Medicine and Science, The University of Nottingham, Sutton Bonington Campus, Leicester, UK; 3 Department of Pathobiology and Population Sciences, The Royal Veterinary College, Hawkshead Lane, North Mymms, Hatfield, Herts AL9 7TA UK; 4 EviVet Evidence-based Veterinary Consultancy, Nottingham, UK

**Keywords:** Animal welfare, Companion animal, Pet ownership, Designer dog, Pedigree, Breed

## Abstract

**Background:**

Demand for intentional crosses of purebred dog breeds, often labelled ‘designer crossbreeds’ (e.g., Labrador Retriever X Poodle, the ‘Labradoodle’), has recently increased in the UK. This study aimed to explore this phenomenon by comparing pre-purchase motivations, pre-purchase and purchase behaviours of UK owners of designer crossbred puppies purchased during 2019-2020 with those of owners of purebred puppies purchased during the same period.

**Results:**

Data were collected in an online cross-sectional survey between November-December 2020. Responses from *n* = 6293 puppies (designer crossbred puppies: *n* = 1575; purebred puppies: *n* = 4718) were analysed. Perceived hypoallergenicity was cited as a motivator for breed/crossbreed choice by almost half of designer crossbreed owners (47.1%), six times more than purebred dog owners (7.86%; odds ratio [OR]: 9.12, 95% CI: 7.70-10.8). Designer crossbred puppies were more likely to have been acquired via a general selling website (e.g., Gumtree; 13.8%) compared to purebred puppies (7.67%; OR: 2.19, 95% CI: 1.77-2.71), or an animal-specific selling websites (e.g., Pets4Homes; 55.7%) compared to purebred puppies (37.4%; OR: 1.89, 95% CI: 1.65-2.17). Designer crossbreed owners were less likely to see their puppy in person prior to purchase than purebred owners (60.4% vs. 67.0%, respectively; OR: 0.74, 95% CI: 0.64-0.85), and at purchase, designer crossbred puppies were less likely to be seen with their mother (73.1% vs. 79.8%, respectively; OR: 0.82, 95% CI: 0.70-0.95), and littermates (67.7% vs. 78.1%, respectively; OR: 0.63, 95% CI: 0.55-0.73). Designer crossbreeds had a significantly higher purchase price, with 25.7% of designer crossbreed puppies costing £2000-£2999 compared to 15.1% of purebred puppies (*X*^2^ = 207.31, *p* <  0.001).

**Conclusions:**

The recent boom in designer crossbreeds in the UK has been fuelled by a desire for perceived hypoallergenic and generally healthy dogs that fit the lifestyles of households with children and limited experience with dogs. Some sought-after traits in designer crossbreeds are misconceptions that risk canine welfare, including relinquishment risk, if owner expectations are not met. Purchasing practices fuelling this boom support irresponsible breeding and selling practices, which combined with reduced pressure for health testing from buyers, may result in a higher disease burden and poorer future welfare for this growing designer dog population.

## Background

The UK has recently witnessed a significant increase in demand for so-called ‘designer crossbreed’ dogs: i.e., purpose-bred crosses originating from two (or sometimes more) defined purebred progenitor breeds [1]. The resultant offspring are often labelled with a portmanteau from progenitor breed names (e.g., the offspring of a Poodle crossed with a Cavalier King Charles Spaniel is labelled a Cavapoo) [2]. The Royal Guide Dog Association of Australia (RGDAA) is often cited as having ‘invented’ the original designer crossbreed, the Australian Labradoodle, in the 1980s by crossing Labrador Retriever guide dogs with non-shedding Poodles in an attempt to create a guide dog that was hypoallergenic. Faced with a paucity of potential fostering candidates due to this new type of guide dog being a crossbreed, ‘creator’ Wally Conran invented the name ‘Labradoodle’ and marketed these crossbreed puppies as a new hybrid dog breed [3]. This marketing campaign was extremely successful and resulted in public attraction towards this allegedly hypoallergenic, non-shedding breed in Australia and internationally [4]. For example, a 2014 study including a random sample of dogs under primary veterinary care in the UK from the VetCompass™ Programme estimated that designer crossbred dogs made up 5.8% of the UK dog population [5], whilst data from the commonly-used online pet-selling website “Pets4Homes” indicated that two designer crossbreeds, the Cockapoo (#4) and Cavapoo (#18), were among the 20 most commonly sold ‘breeds’ in 2020 [6].

Although there are many factors that could be driving the current high demand for designer crossbreeds, there is little published evidence on the motivations of prospective owners towards them. Many common designer crossbreeds are Poodle-crosses, e.g., Labradoodles. These are often marketed based on claims of hypoallergenicity despite little firm supporting evidence for this [4, 7]. Data suggest that levels of dog allergen CanF1 do not differ significantly between homes with ‘hypoallergenic’ and ‘non-hypoallergenic’ breeds, including that of standard, miniature and toy poodles [8] and that classification of Poodle-cross designer breeds as ‘hypoallergenic’ is not supported scientifically [9]. However, until now, is has been unknown whether presumptions of hypoallergenicity are a major driving force behind the acquisition of designer crossbreeds. Given that disparity between owner expectations and the reality of companion animal ownership is a well-recognised risk factor for relinquishment of dogs [10], it is critical to understand this and other motivations for ownership of designer dogs in order to protect the longer-term welfare of these animals.

Choosing which breed to purchase is a multifactorial decision for many prospective dog owners [11, 12]. The desire for the individual puppy to be generally healthy is a commonly stated influence upon breed choice of purebred dogs by prospective owners [11]. It is therefore possible that the puppy-buying public believe that designer crossbreeds are less susceptible to hereditary diseases compared to their progenitor breeds [13]. The hybrid vigour phenomenon suggests that when two distinct breeds within a species are crossed, the resultant offspring will show improved health and welfare compared with the average of the two parents for that characteristic [14]. Recent genetic studies provide some support for this concept. Genotypic data from > 100,000 dogs demonstrated that although mixed breed dogs were significantly more likely to be heterozygous carriers for at least one of nine common largely recessive disease variants when compared to progenitor breeds, purebred dogs were more likely to be phenotypically affected, i.e., carry at least one recessive disease variant in the homozygous state [15]. However, the true level of impact of hybrid vigour upon crossbred dog health has been challenged. It could be reasonably expected that designer crossbreeds exhibit conformational and polygenic disorder occurrence at the midpoint between the values for their progenitor breeds, with any additional health benefits in crossbreds resulting from hybrid vigour effects [16]. A VetCompass study on common disorders in UK dogs reported higher disorder prevalence in purebreds for just 13 of the 84 (15.5%) disorders and syndromes evaluated, compared to crossbred dogs [5], although the severity and duration of these disorders was not assessed. A UK study including data from eye certificates from the British Veterinary Association/Kennel Club/International Sheep Dog Society Eye Schemes found ‘Labradoodles’ exhibited higher levels of multifocal retinal dysplasia (4.6%) than either of their progenitor breeds (0.8 and 0%, respectively) [17]. At present, there is limited evidence-based research outputs that supports either position, and as such, understanding whether designer crossbreed owners are drawn to this type of dog based on health assumptions, is an important step towards understanding their popularity, given it may increase the risk of mismatches between the expectations and realities of owning a designer crossbred dog.

In addition to understanding the motivations for acquiring a designer crossbreed, the behaviours that prospective owners exhibit themselves during the pre-purchase and purchase process are highly relevant to canine welfare. For example, whether owners conducted pre-purchase research and the types of information source consulted [11, 18], identification of breeder(s) and interactions between prospective owners and breeders (e.g., whether a puppy is viewed prior to purchase, and if so, where they are viewed and with which canine relatives, if any) [11] are likely to influence the type of breeder a puppy is sourced from, and thus the welfare of that puppy and its relatives (e.g., its mother and littermates). At present, these decision-making processes have not been reported with regard to designer crossbred dog owners, but are of increasing relevance as the designer crossbred demographic of the UK dog population increases. Rapid population growth for specific subsets of dogs are acknowledged to lead to unpredictable and often severe consequences on the health and welfare of the currently living dogs and also their future potential offspring [19], and thus understanding the recent increase in designer crossbreeds is of timely importance to animal welfare.

The present study aimed to use a cross-sectional analysis of a national survey to characterise and compare:

(i) Pre-purchase motivations;
(ii) Pre-purchase behaviours; and
(iii) Purchase behaviours of UK owners purchasing designer crossbred puppies compared with purebred puppies during 2019 and 2020.

## Materials and methods

### Survey data

This study used data gathered within the ‘Pandemic Puppies’ study [1] collected via an online cross-sectional questionnaire that explored the pre-purchase and purchase motivations and behaviours of puppy buyers in the UK, comparing owners who purchased puppies during the 2020 phase of the COVID-19 Pandemic (23rd March 2020-31st December 2020) vs. the same date frame in 2019. For the present study, data from these periods were used, supplemented by responses from puppy purchases made between January 1st-March 22nd for both years, representing 24 months of acquisition data in total. Full survey design details have been previously published [1]. Briefly, the survey included five sections: (1) General owner demographics, e.g., gender, age, household members, prior dog ownership; (2) General puppy demographics, e.g., breed, sex; (3) Pre-purchase motivations, e.g., factors influencing choice of breed; (4) Pre-purchase behaviours, e.g., research conducted; and (5) Purchase behaviours, e.g., requests for health records, cost of puppy, which parents/relatives of their puppy were seen (if any). The survey was open from 10th November to 31st December 2020, hosted on SurveyMonkey. It was distributed by snowball sampling via a wide range of sources, including social media, the veterinary, canine and general press, and through key stakeholders in canine welfare including the commercial and charity sectors.

Respondents were required to be over 18 years of age, reside in the UK, have brought home a puppy aged under 16 weeks at any date during 2019 or 2020, and have purchased their puppy rather than rehomed or bred the puppy themselves. Where participants had purchased more than one puppy, they were asked to answer for the youngest at the time of the survey. Where littermates had been purchased, owners were asked to answer for the dog whose name came first alphabetically. The raw survey data were exported from SurveyMonkey into Microsoft Excel for manual data cleaning prior to analysis. Responses from duplicate IP addresses (the more complete response was retained), responses without data beyond the consent and inclusion criteria stage, and responses completed by respondents who did not meet the inclusion criteria, were removed prior to analysis. This study received ethical approval from the Social Science Research Ethical Review Board at the Royal Veterinary College (URN: SR2020-0259).

### Spatial analysis

Respondents were asked to provide the first half of their postcodes. These partial postcode data were checked for validity against the Office for National Statistics (ONS) National Statistics Postcode Lookup (NSPL) prior to allocation to one of 12 UK regions [20]. Choropleth maps were produced using ArcGIS 10.2 (Environmental Systems Research Institute) to display the spatial distribution of the designer crossbreed population relative to the study national average across the UK.

### Statistical analysis

Data were imported into IBM SPSS Statistics v27 (SPSS Inc., Chicago, IL, USA) following cleaning in Excel. Designer crossbreeds were defined as first or later generation hybrids of at least two recognised purebreeds that were described using a specific hybrid name by the owner; commonly a portmanteau of the progenitor breeds. Purebred breeds were defined as those having ancestry over many generations of the same breed, which are recognised as such by The Kennel Club [21] or other international kennel clubs, and were identified in the dataset by this recognised breed name. Mixed breeds were categorised as non-purebred animals that were not described with a specific hybrid name (e.g., ‘Crossbreed’, ‘Mixed breed’) and were excluded from further analysis in the current study.

Categorical variables describing pre-purchase motivations and behaviour (e.g., factors influencing breed choice, pre-purchase viewing of puppies) and purchasing behaviours (e.g., where the puppy was collected from) were compared between designer crossbred puppies and purebred puppies at the univariable level using chi-squared (*X*^2^) analysis. Variables that were liberally associated with designer crossbred puppy ownership at the univariable level (*p* <  0.2) were taken forward to separate multivariable binary logistic regression modelling with designer crossbreed (yes/no) as the binary outcome variable. Owner age, UK region, acquisition year (to account for the ‘Pandemic Puppy’ effect [1]), whether children were present in the household and whether the owner had prior dog ownership experience were included as a fixed set of covariables for all models, due to these variables having been found to influence puppy purchasing in this dataset (for the latter four variables [1]) and owner age influencing puppy purchasing in a previous study, particularly around purebred dog acquisition [22]. Although initially considered as a covariate due to its influence on acquisition preferences in previous studies [22], owner gender was not included in the final models due to poor model fit. The Hosmer-Lemeshow test was used to evaluate the quality of the model fit. Statistical significance was set at the 5% level.

## Results

In total, *n* = 7545 responses were obtained to the survey. Following cleaning, the remaining valid sample (*n* = 6293), included *n* = 1575 designer crossbred puppies (25.03%) and *n* = 4718 purebred puppies (74.97%) which were taken forward for analysis.

### Spatial analysis

**Fig. 1:**
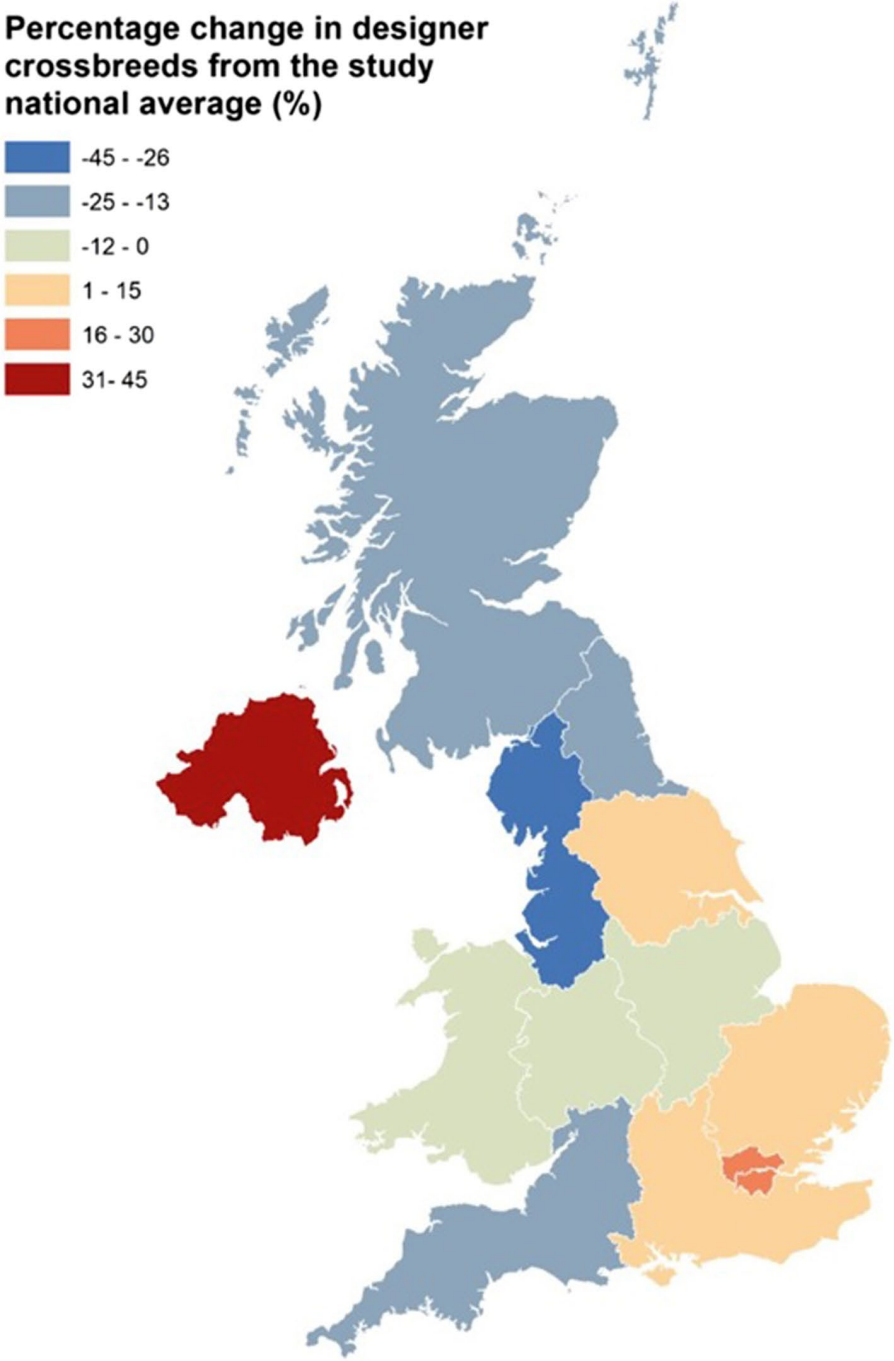
Choropleth map showing regional percentage differences from the overall study national percentage of designer crossbred puppies in the UK in 2019-2020 (25.03%)

All UK regions were represented in the study sample, with geographical distribution of designer crossbred puppies shown in Fig. 1. Owners of designer crossbred puppies were significantly more likely to live in London (designer crossbred: 12.5% vs. purebred: 8.74%, *X*^2^ = 39.44, *p* <  0.001) than owners of purebred puppies.

### Owner demographics and lifestyle

Overall respondents were predominantly female, with no difference in gender distribution between designer cross and purebred puppy owners (female, designer crossbred: 91.8%, *n* = 1249, vs. purebred: 90.6%; *n =* 3713, *X*^2^ = 2.44, *p* = 0.486). The most common age group of owners was 45-54 years old (designer crossbred: 27.9%, *n =* 380, vs. purebred: 24.0%, *n =* 984,), followed by 25-34 years old (designer crossbred: 22.5%, *n =* 306, vs. purebred: 24.0%, *n =* 985,); owners of designer crosses were more likely to be aged between 35 and 54 (51.7%) compared to purebred puppy owners (44.6%) (*X*^2^ = 23.68, *p* <  0.001). The majority of respondents described themselves as the primary carer for their puppy (designer crossbred: 57.1% vs. purebred: 60.2%); with no significant difference between owners of designer crosses and purebred puppies (*X*^2^ = 7.19, *p* = 0.126).

### Prior experience of dog ownership

Owners of designer crossbred puppies were less likely to have previously owned/co-owned a dog before their current puppy, with 46.1% (*n =* 627) of designer crossbred puppy owners having previous dog ownership experience compared to 67.6% (*n =* 2769) of purebred puppy owners (*X*^2^ = 213.73, *p* <  0.001). Within designer dog households, all members of the household were more likely to be a first-time dog owner compared to households that had purchased a purebred puppy (designer crossbred: 38.0%, *n =* 517, vs. purebred: 20.7%, *n =* 845; *X*^2^ = 213.73, *p* <  0.001). Owners of designer crossbred puppies were less likely to have grown up with a dog (61.2%, *n =* 832) compared to purebred puppy owners (70.3%, *n =* 2875; *X*^2^ = 38.85, *p* <  0.001). Owners of designer crosses were less likely to be employed in the animal care sector (e.g., veterinary surgeons, veterinary nurses, dog trainers; 5.05%, *n =* 67) compared to purebred puppy owners (15.1%, *n =* 620; *X*^2^ = 96.93, *p* <  0.001).

### Household demographics

Designer crossbred puppies were less likely to live in an adult-only home (designer crossbred: 50.8%, *n =* 691 vs. purebred: 59.9%, *n =* 2450; *X*^2^ = 51.08, *p* <  0.001), and were less likely to live in a home where the respondent lived alone (designer crossbred: 7.58%, *n =* 103, vs. purebred: 8.94%, *n =* 366; *X*^2^ = 51.08, *p* <  0.001). Designer crossbred puppies were significantly more likely to be the only dog in the household (82.9%, *n =* 1084) compared to purebred puppies, (60.7%, *n =* 2414; *X*^2^ = 234.58, *p* <  0.001). There was no significant difference between designer crossbred puppies and purebred puppies that had access to either a shared (3.14% vs. 2.63%, respectively) or private garden (95.0% vs. 96.0%, respectively) or yard (1.65% vs. 1.19%, respectively; *X*^2^ = 2.64, *p* = 0.450).

### Puppy demographics

The five most frequent designer crossbreeds in the study were the Cockapoo (32.1% of all designer crossbred dogs, *n =* 506), Labradoodle (11.6%, *n =* 183), Cavapoo (9.10%, *n =* 144), Sprocker (6.00%, *n =* 95), and Goldendoodle (3.20%, *n =* 50). The five most frequent purebred breeds were the Labrador Retriever (14.0% of all purebred dogs, *n =* 662), Cocker Spaniel (9.70%, *n =* 459), Miniature Smooth-Haired Dachshund (6.40%, *n =* 300), Border Collie (5.20%, *n =* 246), and Border Terrier (4.40%, *n =* 206).

Whilst the majority of puppies in the overall study were insured (83.6% overall), a higher proportion (87.6%, *n =* 1370) of the designer crossbred puppy population were insured compared to the purebred puppy population (82.5%, *n =* 3894; *X*^2^ = 47.11, *p* <  0.001). Sex distribution did not differ significantly between designer crossbred puppies and purebred puppies (50.0% female vs. 46.8% female, respectively; *X*^2^ = 3.44, *p* = 0.064).

### Pre-purchase motivations

Companionship for the owner was cited as the most common reason to want to purchase a puppy for both designer crossbreed puppy and purebred puppy owners (designer crossbreed: 68.1% vs. purebred: 63.5%), followed by exercise encouragement (designer crossbreed: 63.3% vs. purebred: 48.1%). After accounting for cofounding at the multivariable level, both of these reasons for wanting to purchase a dog had significantly higher odds in the owners of designer crosses compared to owners of purebred puppies (companionship for themselves (odds ratio [OR]: 1.23, 95% CI: 1.06-1.42, *p* = 0.006); exercise encouragement for the owner/their family (OR: 1.52, 95% CI: 1.32-1.74, *p* = 0.006)) (Table 1). Designer crossbreed owners also had a higher odds of seeking a dog to improve their/their family’s mental health (OR: 1.54 95% CI: 1.34-1.77, *p* <  0.001) compared to purebred puppy owners (Table 1). Conversely, designer crossbreed owners had lower odds of wanting to purchase a dog due to the loss of a previous dog in the household (OR: 0.76, 95% CI: 0.63-0.91, *p* = 0.002), companionship for other dogs in the household (OR: 0.67, 95% CI: 0.55-0.82, *p* <  0.001), for a working role (e.g., assistance dog) (OR: 0.45, 95% CI: 0.32-0.61, *p* <  0.001) or for a specific non-working role (e.g., showing) (OR:0.21, 95% CI: 0.12-0.37, *p* <  0.001) compared with purebred dog owners (Table 1).

**Table 1:** Multivariable logistic regression modelling of reasons why owners wanted to purchase a dog (*n =* 6281), with comparisons between designer crossbreed puppy owners (*n =* 1568) and purebred puppy owners (*n =* 4713) in the UK. Significant associations (*p* <  0.05) are emboldened. ^a^Footnote

Reason for wanting to purchase a dog (***n*** = 6281)	Designer crossbred % (***n*** = 1568)	Purebred % (***n*** = 4713)	Odds Ratio	95% CI^**b**^	***p*** value
Companionship for myself	68.1	63.5	**1.23**	**1.06-1.42**	**0.006**
To encourage myself/my family to walk and exercise	63.3	48.1	**1.52**	**1.32-1.74**	**< 0.001**
To improve my/my family’s mental health	55.2	40.6	**1.54**	**1.34-1.77**	**< 0.001**
Companionship for other adult(s) in my household	38.7	35.2	1.06	0.92-1.21	0.421
Companionship for my children	30.7	19.8	**1.28**	**1.02-1.59**	**0.032**
To keep me/my family busy	26.9	21.9	1.16	0.10-1.36	0.052
Due to the loss of a previous dog in my household	20.7	32.4	**0.76**	**0.63-0.91**	**0.002**
Companionship for my other dog(s)	12.4	25.1	**0.67**	**0.55 – 0.82**	**< 0.001**
As a working dog for a specific role (e.g., gundog, security, sniffer/tracking, herding, medical detection, assistance/therapy dog)	3.70	10.4	**0.45**	**0.32-0.61**	**< 0.001**
For a specific non-working role (e.g., dog sports, showing, etc.)	1.02	6.98	**0.21**	**0.12-0.37**	**< 0.001**

^a^Fixed covariables used in this model were owner age, UK region, acquisition year (2019 or 2020), whether children were present in the household and whether the owner had prior dog ownership experience

^b^Confidence interval

When deciding which breed or crossbreed to purchase, the most commonly sought-after breed/crossbreed characteristics by both designer crossbred and purebred puppy owners were good companionship (designer crossbred: 73.7% vs. purebred: 69.4%, *X*^2^ = 10.21, *p* <  0.001), and bodysize which was suited to owner lifestyle (designer crossbred: 74.8% vs. purebred: 59.1%, *X*^2^ = 122.36, *p* <  0.001) (Table 2). After accounting for cofounding at the multivariable level owners of designer crossbreeds had significantly higher odds compared to owners of purebred puppies of seeking out a breed/crossbreed size suited to the owners’ lifestyle (OR: 1.86, 95% CI: 1.60-2.16, *p* <  0.001). In addition, designer crossbreed puppy owners had higher odds of seeking a breed/crossbreed that they believed to be hypoallergenic (OR: 9.12, 95% CI: 7.70-10.79, *p* <  0.001), generally healthy (OR: 2.05, 95% CI: 1.79-2.34, *p* <  0.001), that was easy to train (OR:1.92, 95% CI: 1.68-2.19, *p* <  0.001), was good with children (OR: 1.46, 95% CI: 1.25-1.69, *p* <  0.001), that friends or family currently owned (OR: 1.23, 95% CI: 1.06-1.44, *p* = 0.006), and had an affordable purchase price (OR: 1.93, 95% CI: 1.55-2.39, *p* <  0.001) compared with purebred dog owners (Table 2). Conversely, owners of designer crossbreeds had lower odds of seeking out a particular breed/crossbreed they had previous ownership experience of (OR: 0.22, 95% CI: 0.18-0.27, *p* <  0.001), childhood experiences of (OR: 0.33, 95% CI: 0.27-0.41, *p* <  0.001), or a breed/crossbreed with low grooming needs (OR: 0.70, 95% CI: 0.58-0.85, *p* <  0.001), or working ability (OR: 0.29, 95% CI: 0.22-0.39, *p* <  0.001) compared to purebred dog owners (Table 2).

**Table 2:** Multivariable logistic regression modelling of characteristics sought by owners when selecting a puppy to purchase (*n =* 6175), with comparisons between designer crossbred puppy owners (*n =* 1545) and purebred puppy owners (*n =* 4630) in the UK. Significant associations (*p* <  0.05) are emboldened. ^a^Footnote

Breed/crossbreed characteristic (***n*** = 6175)	Designer crossbred % (***n*** = 1545)	Purebred % (***n*** = 4630)	Odds Ratio	95% CI^**b**^	***p*** value
Size is suited to my lifestyle	74.8	59.1	**1.86**	**1.60-2.16**	**< 0.001**
Good companion	73.7	69.4	1.15	0.99-1.34	0.062
Generally healthy breed/crossbreed	61.2	42.3	**2.05**	**1.79-2.34**	**< 0.001**
Good with children	56.0	42.5	**1.46**	**1.25-1.69**	**< 0.001**
Easy to train	54.3	36.4	**1.92**	**1.68-2.19**	**< 0.001**
Hypoallergenic	47.1	7.86	**9.12**	**7.70-10.8**	**< 0.001**
Appearance/looks	42.7	38.9	1.11	0.97-1.27	0.138
Exercise encouragement	34.4	31.2	1.06	0.92-1.22	0.405
Friends or family currently own this breed/crossbreed	28.9	21.8	**1.23**	**1.06-1.44**	**0.006**
Long life expectancy	13.5	15.1	0.95	0.78-1.14	0.564
Low grooming needs	13.1	16.5	**0.70**	**0.58-0.85**	**< 0.001**
Affordable purchase cost of puppies	12.9	6.80	**1.93**	**1.55-2.39**	**< 0.001**
I’ve owned this breed/crossbreed before	12.8	42.9	**0.22**	**0.18-0.27**	**< 0.001**
Affordable cost of upkeep	9.45	7.04	1.22	0.97-1.55	0.091
I grew up with or had childhood experiences with this breed/crossbreed	8.61	21.8	**0.33**	**0.27-0.41**	**< 0.001**
Popularity of the breed/crossbreed	6.60	3.76	**1.45**	**1.07-1.95**	**0.016**
Low exercise requirements	5.83	5.33	0.94	0.70-1.26	0.692
Working ability of this breed/crossbreed	4.92	18.1	**0.29**	**0.22-0.39**	**< 0.001**

^a^Fixed covariables used in this model were owner age, UK region, acquisition year (2019 or 2020), whether children were present in the household and whether the owner had prior dog ownership experience

^b^Confidence interval

The most common characteristics owners of both designer crossbred and purebred puppy owners sought out in a breeder was a breeder who they felt were trustworthy (designer crossbreed: 76.6% vs. purebred: 79.7%), and that performed the health tests for the breed/crossbreed that they wanted (designer crossbreed: 59.3% vs. purebred: 66.2%). After accounting for cofounding in multivariable analyses, owners of designer crossbreeds had a higher odds of seeking out a breeder that lived within the distance they were willing to travel (OR: 1.63, 95% CI: 1.42-1.86, *p* <  0.001) and had availability of puppies at the desired time (OR: 1.34, 95% CI: 1.21-1.59, *p* <  0.001) compared with purebred dog owners (Table 3). In contrast, owners of designer crossbreed puppies had lower odds of seeking a breeder that they felt was trustworthy (OR: 0.85, 95% CI: 0.72-0.10, *p* = 0.043), that was a member of the Kennel Club Assured Breeders Scheme (OR: 0.13, 95% CI: 0.10-0.18, *p* <  0.001), that bred the particular colour of puppies they wanted to purchase (OR: 0.85, 95% CI: 0.72-0.10, *p* = 0.043), or that performed the relevant health tests for their breed/crossbreed (OR: 0.75, 95% CI: 0.65-0.86, *p* <  0.001) compared to purebred puppy owners (Table 3).

**Table 3:** Multivariable logistic regression modelling of characteristics of breeders that prospective owners sought out (*n =* 6037), with comparisons between designer crossbred puppy owners (*n =* 1511) and purebred puppy owners (*n =* 4526) in the UK. Significant associations (*p* <  0.05) are emboldened. ^a^Footnote

Breeder characteristic (***n*** = 6037)	Designer crossbred % (***n*** = 1511)	Purebred % (***n*** = 4526)	Odds Ratio	95% CI^**b**^	***p*** value
Someone I felt was trustworthy	76.6	79.7	**0.85**	**0.72-1.00**	**0.043**
They performed health tests for the breed/crossbreed I wanted	59.3	66.2	**0.75**	**0.65-0.86**	**< 0.001**
Lived within the distance I was willing to travel	48.0	36.2	**1.63**	**1.42-1.86**	**< 0.001**
Availability of puppies at the time I wanted	45.9	37.2	**1.39**	**1.21-1.59**	**< 0.001**
They would allow me to see the puppies’ father (sire)	40.4	42.8	0.95	0.83-1.09	0.498
Reasonably priced puppies	37.1	35.0	1.13	0.98-1.29	0.093
Bred the colour of the breed/crossbreed I wanted to purchase	20.3	23.3	**0.85**	**0.72-1.00**	**0.043**
A member of the Kennel Club Assured Breeders Scheme	5.29	27.4	**0.13**	**0.10-0.18**	**< 0.001**
The dogs they bred from had been awarded prizes at dog shows	1.46	12.0	**0.13**	**0.08-0.21**	**< 0.001**

^a^Fixed covariables used in this model were owner age, UK region, acquisition year (2019 or 2020), whether children were present in the household and whether the owner had prior dog ownership experience

^b^Confidence interval

### Pre-purchase Behaviours of Owners

Pre-purchase research was more common amongst designer crossbreed owners compared to purebred puppy owners (designer crossbreed: 73.0% vs. purebreed: 48.6%; *X*^2^ = 322.62, *p* <  0.001). A lower proportion of designer crossbreed owners considered themselves to already be experienced dog owners who therefore did not need to undertake any pre-purchase research (designer crossbreed: 23.0% vs. purebreed: 48.9%; *X*^2^ = 322.62, *p* <  0.001). Only 4.02% of designer crossbred puppy owners and 2.49% of purebred puppy owners reported undertaking no pre-purchase research at all (Table 4).

**Table 4:** Multivariable logistic regression modelling of sources of information used when researching dog ownership and/or which breed/crossbreed to purchase prior to purchasing a puppy (*n =* 3798), with comparisons between designer crossbred puppy owners (*n =* 1155) and purebred puppy owners (*n =* 2643) in the UK. Significant associations (*p* <  0.05) are emboldened. ^a^Footnote

Source (***n*** = 3798)	Designer crossbred % (***n*** = 1155)	Purebred % (***n*** = 2643)	Odds Ratio	95% CI^**b**^	***p*** value
A breed/crossbreed-specific online resource (e.g., website/forum)	75.2	50.4	**2.81**	**2.35-3.35**	**< 0.001**
Talking to friends or family who own or had owned a dog	74.3	57.8	**1.66**	**1.38-2.00**	**< 0.001**
Social media sites, e.g., Facebook, Instagram	52.8	42.7	**1.42**	**1.21-1.67**	**< 0.001**
An animal charity website, e.g., Dogs Trust, RSPCA, PDSA, etc.	52.6	34.6	**1.94**	**1.64-2.29**	**< 0.001**
Talking to a dog breeder	42.9	53.4	**0.60**	**0.51-0.70**	**< 0.001**
The Kennel Club website	37.5	59.4	**0.34**	**0.29-0.40**	**< 0.001**
Book(s)	35.4	33.1	0.91	0.77-1.08	0.269
My veterinary professional (e.g., veterinary surgeon, veterinary nurse)	10.2	12.4	0.83	0.64-1.07	0.158
Other digital sources (e.g., articles on the internet, TV shows)	4.07	2.84	**1.53**	**1.00-2.33**	**0.049**

^a^Fixed covariables used in this model were owner age, UK region, acquisition year (2019 or 2020), whether children were present in the household and whether the owner had prior dog ownership experience

^b^Confidence interval

After accounting for cofounding in multivariable analyses, it was identified that for those participants that did complete pre-purchase research, designer crossbreed owners had higher odds of using online resources such as breed/crossbreed specific online resources (e.g., websites/forums) (OR: 2.81, 95% CI: 2.35-3.35, *p* <  0.001), social media sites (e.g., Facebook/Instagram) (OR: 1.42, 95% CI: 1.21-1.67, *p* <  0.001), animal charity websites (e.g., RSPCA, Dogs Trust) (OR: 1.94, 95% CI: 1.64-2.29, *p* <  0.001), and other digital sources (e.g., internet articles) (OR: 1.53, 95% CI: 1.00-2.33, *p* = 0.049) and speaking to family/friends that already owned a dog prior to purchase (OR: 1.66, 95% CI: 1.38-2.00, *p* <  0.001) compared with purebred dog owners (Table 4). In contrast, designer crossbred puppy owners had significantly lower odds of conducting pre-purchase research using the Kennel Club website (OR: 0.34, 95% CI: 0.29-0.40, *p* = 0.339) or to have spoken directly to a breeder (OR: 0.60, 95% CI: 0.51-0.70, *p* <  0.001) (Table 4).

The most common places that both designer crossbred and purebred puppy owners found their dogs were animal specific selling websites (e.g., Pets4Homes) (designer crossbred: 55.7% vs. purebred: 37.4%) or through a breeder they already knew (designer crossbred: 12.3% vs. purebred: 24.8%) (Table 5). Designer crossbred puppy owners were 18.3% more likely to have found their puppy using an animal specific selling website when compared to purebred puppy owners (Table 5), with multivariable analysis finding they had 1.89 higher odds of finding their puppy this way compared to purebred puppy buyers (CI: 1.65-2.17, *p* < 0.001). In addition, designer crossbred puppy owners had 2.19 higher odds of finding their puppy via a general selling website (e.g., Gumtree; CI: 1.77-2.71, *p* < 0.001) compared to purebred dog owners (Table 5). Designer crossbred puppy owners had lower odds of finding their puppy through recommendations from another breeder/stud dog owner (OR: 0.13, 95% CI: 0.05-0.35, *p* < 0.001), recommendations from acquaintances (e.g., colleagues, friends, family, animal professionals) (OR: 0.35, 95% CI: 0.18-0.68, *p* = 0.002), through breeders they already knew (OR: 0.54, 95% CI: 0.44-0.64, *p* < 0.001) or using the Kennel Club ‘Find a Puppy’ search tool (OR: 0.10, 95% CI: 0.07-0.14, *p* < 0.001) compared to purebred puppy owners (Table 5).

**Table 5:** Multivariable logistic regression modelling of places owners found their puppy (*n =* 6034) with comparisons between designer crossbred puppy owners (*n =* 1509) and purebred puppy owners (*n =* 4525). Significant associations (*p* < 0.05) are emboldened. ^a^Footnote

Places owners found their puppy (***n*** = 6034)	Designer crossbred % (***n*** = 1509)	Purebred % (***n*** = 4525)	Odds Ratio	95% CI^**b**^	***p*** value
An animal specific selling website, e.g., Pets4Homes, Champdogs	55.7	37.4	**1.89**	**1.65-2.17**	**< 0.001**
A general selling website, e.g., FreeAds, Gumtree, Preloved	13.8	7.67	**2.19**	**1.77-2.71**	**< 0.001**
I already knew the breeder (e.g., colleague, friends, family, repeat purchase)	12.3	24.8	**0.53**	**0.44-0.64**	**< 0.001**
A social media breed/crossbreed specific group	9.54	8.42	1.15	0.91-1.44	0.245
The Kennel Club website ‘Find A Puppy’ search	2.32	17.5	**0.01**	**0.07-0.14**	**< 0.001**
Recommendation from someone who is not a colleague, friend, family member or animal professional	0.66	2.03	**0.35**	**0.18-0.68**	**0.002**
Recommendation from another breeder/stud dog owner	0.33	2.21	**0.13**	**0.05-0.35**	**< 0.001**

^a^Fixed covariables used in this model were owner age, UK region, acquisition year (2019 or 2020), whether children were present in the household and whether the owner had prior dog ownership experience

^b^Confidence interval

**Fig. 2:**
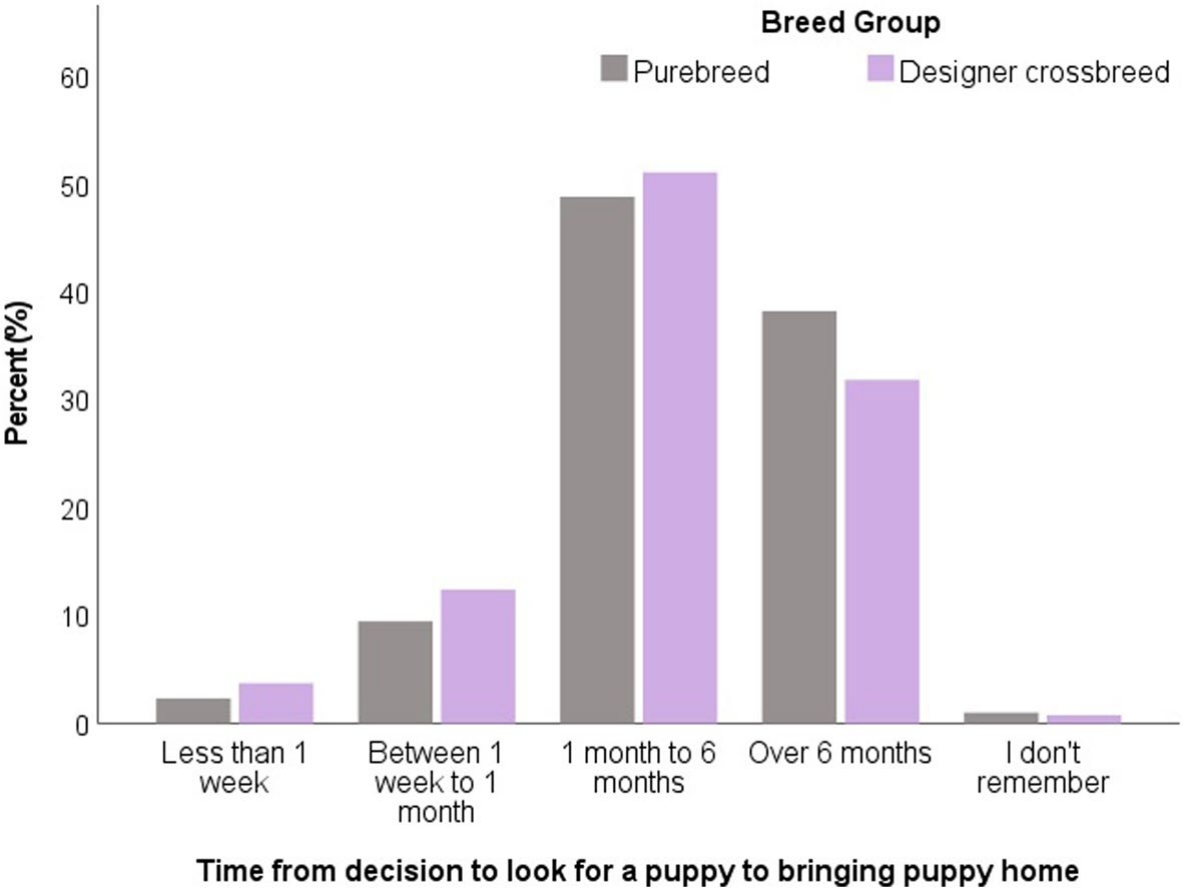
Interval from deciding to look for a puppy to acquisition (bringing puppy home) (*n =* 6168) with comparison between designer crossbred puppy owners (*n =* 1545) and purebred puppy owners (*n =* 4625) in the UK

The time interval between prospective owners’ initial decision to look for a puppy to when they brought their puppy home was most commonly between one to 6 months for both designer crossbred puppy owners (51.1%) and purebred puppy owners (48.9%). Owners of designer crosses were more likely to spend less than 1 week (designer crossbred: 3.76% vs. purebred: 2.34%) and between 1 week to 1 month (designer crossbred: 12.4% vs. purebred: 9.49%; *X*^2^ = 33.30, *p* < 0.001) from the decision to look for a puppy and when their puppy was brought home compared to purebred puppy owners (Fig. 2).

**Fig. 3:**
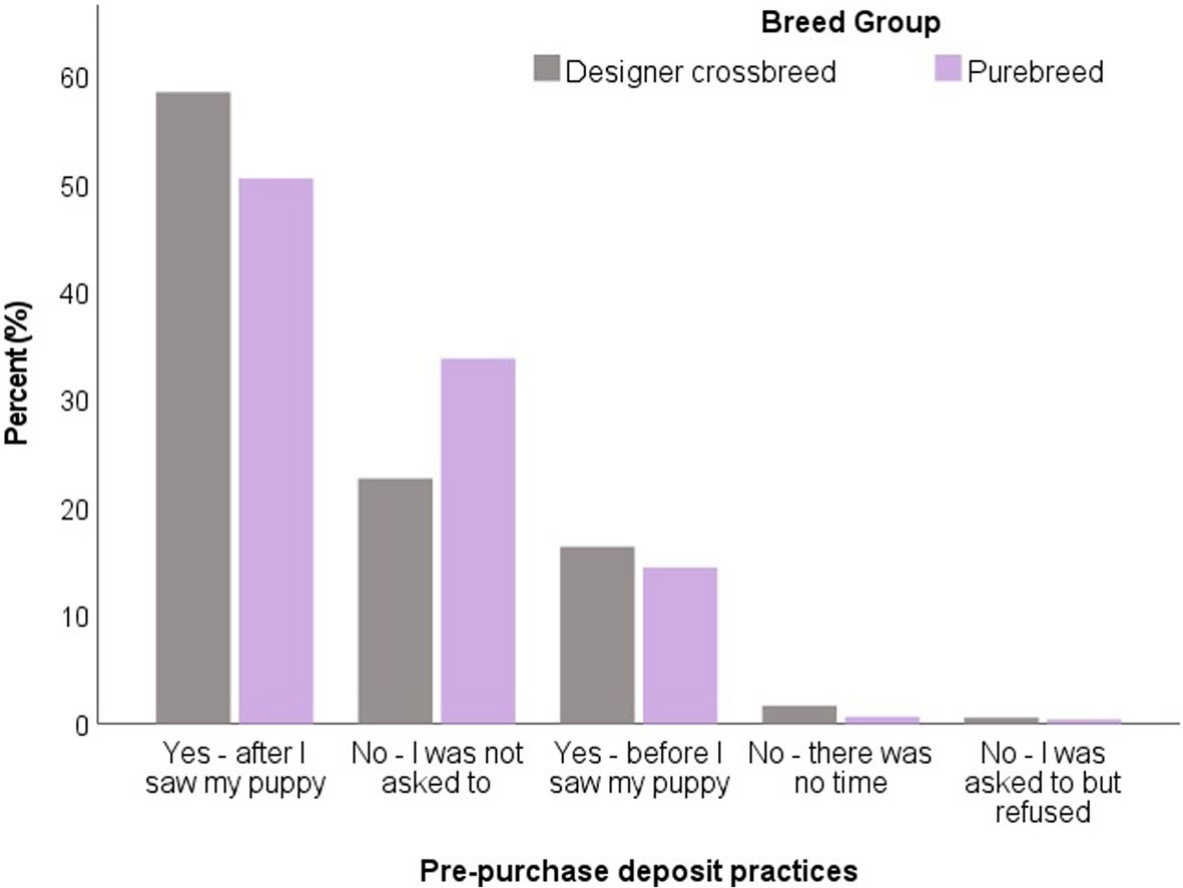
Deposit practices to secure puppies prior to purchase (*n =* 5042) with comparison between designer crossbred puppy owners (*n =* 1252) and purebred puppy owners (*n =* 3790) in the UK

Designer crossbred puppy owners were more likely to be asked to put down a deposit on their puppy compared to purebred puppy owners, both before seeing their puppy (designer crossbred: 16.4% vs. purebred: 14.4%) or after seeing their puppy (designer crossbred: 58.4% vs. purebred: 50.3%; *X*^2^ = 63.84, *p* < 0.001) (Fig. 3).

The most common pre-purchase viewing practices for owners of both designer crossbred and purebred puppies were visiting the breeder’s property in person (designer crossbred: 60.4% vs. purebred: 67.0%) and seeing photos/pre-recorded video of their puppy (designer crossbred: 45.6% vs. purebred: 45.8%) (Table 6). After accounting for confounding in the multivariable analysis, owners of designer crosses had significantly lower odds of visiting the breeder’s property in person when compared with purebred puppy owners (OR: 0.74, 95% CI: 0.64-0.85, *p* < 0.001), or bringing their puppy home on the same day they were viewed, due to the purchase being a rapid decision (OR: 0.41, 95% CI: 0.29-0.57, *p* < 0.001).

**Table 6:** Multivariable logistic regression modelling of puppy viewing practices prior to the date the puppy was brought home (*n =* 5964) with comparison between designer crossbred puppy owners (*n =* 1494) and purebred puppy owners (*n =* 4470) in the UK. Significant associations (*p* < 0.05) are emboldened. ^a^Footnote

Viewed prior to the date they were brought home (***n*** = 5964)	Designer crossbred % (***n*** = 1494)	Purebred % (***n*** = 4470)	Odds Ratio	95% CI^**b**^	***p*** value
Yes – visited the breeder’s property in person	60.4	67.0	**0.74**	**0.64-0.85**	**< 0.001**
No – I wanted to see my/our puppy, but the breeder refused	48.0	0.76	0.67	0.33-1.33	0.250
Yes – saw my/our puppy on a live video call with their breeder	24.5	21.4	1.08	0.92-1.27	0.327
No – the purchase was a rapid decision, so my puppy was brought home on the same day they were viewed	4.95	2.42	**0.41**	**0.29-0.57**	**< 0.001**

^a^Fixed covariables used in this model were owner age, UK region, acquisition year (2019 or 2020), whether children were present in the household and whether the owner had prior dog ownership experience

^b^Confidence interval

Whilst the majority of owners purchased their first-choice breed/crossbreed (89.3% overall) owners of designer crossbred puppies were less likely to purchase their first-choice compared to purebred puppy owners (designer crossbred: 79.7% vs. purebred: 92.5%; *X*^2^ = 220.87, *p* < 0.001) (Table 7). The most common reasons given by designer crossbred owners for not purchasing their first-choice breed/crossbreed were that they could not find a seller that had puppies of their first-choice breed/crossbreed available at the time they wanted (5.52%) or that they did not have a specific first-choice breed/crossbreed in mind (4.68%) (Table 7).

**Table 7:** Purchase levels of first-choice breeds/crossbreeds and reasons for not purchasing their first-choice breed (*n =* 6138) with comparison between designer crossbred puppy owners (*n =* 1539) and purebred puppy owners (*n =* 4599) in the UK

First-choice breed? (***n*** = 5964)	Designer crossbred % (***n*** = 1539)	Purebred % (***n*** = 4599)
Yes, my puppy/dog is the breed/crossbreed that was my first-choice	79.7	92.5
No, I could not find a seller that had puppies available at the time for my first-choice breed/crossbreed	5.52	2.07
No, I/we didn’t have a specific choice in mind	4.68	1.37
No, puppies of my first-choice breed/crossbreed were too expensive	3.64	1.35
No, I/we changed our mind	1.69	0.59
No, I could not find a breeder I felt happy buying a puppy from for my first-choice breed/crossbreed	1.23	0.98
No, but I had several breeds (> 3) that I was interested in and got one of those	0.97	0.30
No, puppies of my first-choice breed/crossbreed were too far away	0.71	0.02
No, but I had several breeds (≤ 3) I was interested in and got one of them	0.71	0.15
No, I wanted a rescue	0.65	0.41
No, our planned purchase of our first-choice fell through	0.26	0.17

### Purchasing Behaviours of Owners

The most common locations for both designer cross and purebred puppy owners to collect their puppy were inside the breeder’s property (designer crossbred: 59.4% vs. purebred: 61.8%) or outside the breeder’s property (designer crossbred: 24.8% vs. purebred: 21.4%) (Table 8). After accounting for cofounding in multivariable analyses, there were no significant differences between the locations at which designer crossbred puppy owners and purebred puppy owners received their puppy on the day they were brought home (Table 8).

**Table 8:** Multivariable logistic regression modelling of locations owners received their puppy (*n =* 6037) with comparisons between designer crossbred puppy owners (*n =* 1511) and purebred puppy owners (*n =* 4526) in the UK. ^a^Footnote

Location (***n*** = 6037)	Designer crossbred % (***n*** = 1511)	Purebred % (***n*** = 4526)	Odds Ratio	95% CI^**b**^	***p*** value
The breeder’s property – from inside their home	59.4	61.8	0.96	0.18-0.26	0.595
The breeder’s property – from outside their home, e.g., doorstep, garden	24.8	21.4	1.11	0.95-1.31	0.194
A car park	1.32	0.68	1.73	0.92-3.26	0.090

^a^Fixed covariables used in this model were owner age, UK region, acquisition year (2019 or 2020), whether children were present in the household and whether the owner had prior dog ownership experience

^b^Confidence interval

Designer crossbreed owners were less likely to be provided with health testing results for their puppies’ parents by their puppies’ breeder, for both DNA (genetic) tests (*X*^2^ = 40.46, df = 3, *p* < 0.001) and veterinary screening tests (e.g., hips, elbows, knees, eyes, respiratory testing; *X*^2^ = 69.61, df = 3, *p* < 0.001) at the univariable level. These differences were largely driven by designer crossbreed buyers being less likely to ask their breeder about health testing (−8.2%; veterinary screening tests) than purebred puppy buyers, or not believing that there are any health tests available for their breed/crossbreed (−4.4%; DNA tests) than purebred puppy buyers (Table 9).

**Table 9:** Owners’ requests for information related to health testing of their puppies’ parents with comparison between purebred and designer crossbred puppy owners in the UK

Test Type	Request and Provision of information	Breed Group	
		Designer crossbred % (***n*** = 1235)	Purebred % (***n*** = 3718)
Results of DNA (genetic) tests	Yes, and they provided me with it	38.2	46.0
	Yes, but they could not provide me with it	3.7	3.7
	No, I didn’t ask about this	47.5	44.2
	No, I do not believe there are any tests available for my puppy’s breed/crossbreed	10.5	6.1
Results of veterinary screening tests (e.g., hips, elbows, knees, eyes, respiratory testing)	Yes, and they provided me with it	41.2	54.1
	Yes, but they could not provide me with it	5.0	4.2
	No, I didn’t ask about this	44.3	36.1
	No, I do not believe there are any tests available for my puppy’s breed/crossbreed	9.6	5.6

On the day of collection, both designer crossbred and purebred puppies were most commonly seen with their mother (dam, designer crossbred: 73.1% vs. purebred: 79.8%), followed by their littermates (designer crossbred: 67.7% vs. purebred: 78.1%) (Table 10). However, multivariable logistic regression identified that, after accounting for cofounding, designer crosses were at 0.82 lower odds of being seen with their mother (dam) (95% CI: 0.70-0.95, *p* = 0.009), 0.63 lower odds of being seen with their littermates (95% CI: 0.55-0.73, *p* < 0.001), and 0.43 lower odds of being seen with other adult dogs that were claimed to be relatives (e.g., grandparents, older siblings) (95% CI: 0.30-0.61, *p* < 0.001) compared to purebred puppies (Table 10). In contrast, designer crossbred puppies had higher odds of being seen with other puppies (unsure if they were littermates) (OR: 1.41, 95% CI: 1.00-1.99, *p* = 0.047).

**Table 10:** Multivariable logistic regression modelling of other dogs that were seen at the seller’s premises on the day of purchase of the puppy (*n =* 5904) with comparisons between designer crossbred puppy owners (*n =* 1478) and purebred puppy owners (*n =* 4426) in the UK. Significant associations (*p* < 0.05) are emboldened. ^a^Footnote

Other dogs and their relationship to the purchased puppy (***n*** = 5904)	Designer crossbred % (***n*** = 1478)	Purebred % (***n*** = 4426)	Odds Ratio	95% CI^**b**^	***p*** value
Their mother (dam)	73.1	79.8	**0.82**	**0.70-0.95**	**0.009**
Their littermates	67.7	78.1	**0.63**	**0.55-0.73**	**< 0.001**
Another dog(s) they were not related to (e.g., another breed)	35.8	28.6	0.97	0.83-1.12	0.649
Their father (sire)	21.5	24.8	0.51	0.81-1.11	0.949
I only saw my/our puppy	13.7	9.51	1.21	0.99-1.49	0.062
Adult dog(s) they were related to (e.g., aunts, grandparents, older siblings)	3.00	7.41	**0.43**	**0.30-0.61**	**< 0.001**
Other puppies (unsure if they were littermates)	4.19	2.96	**1.41**	**1.00-1.99**	**0.047**

^a^Fixed covariables used in this model were owner age, UK region, acquisition year (2019 or 2020), whether children were present in the household and whether the owner had prior dog ownership experience

^b^Confidence interval

**Fig. 4:**
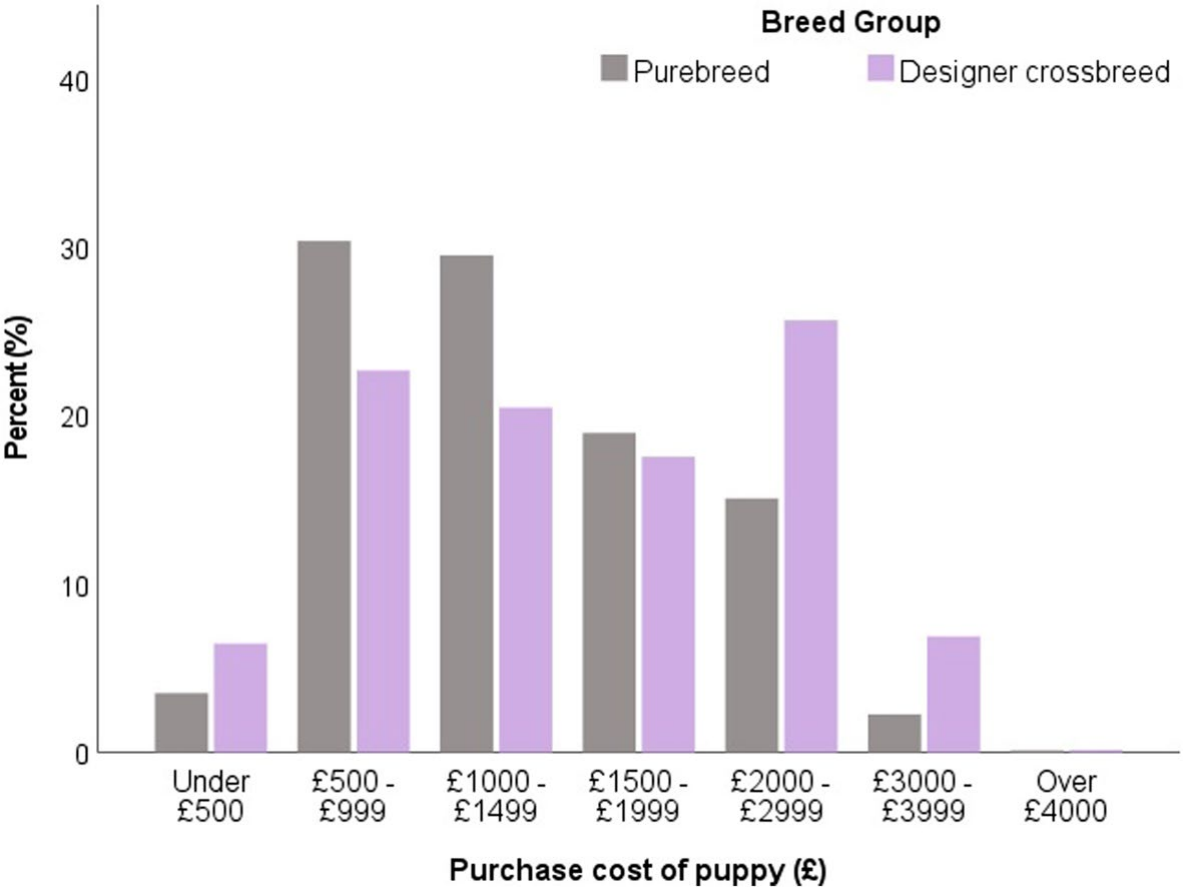
Purchase price of puppies (excluding any associated purchases, e.g., food, collar, bowls) (*n =* 5542) with comparison between designer crossbred puppy owners (*n =* 1405) and purebred puppy owners (*n =* 4137) in the UK

Both designer crossbred and purebred puppies were most commonly reported to be aged between seven to 8 weeks old when they were purchased (designer crossbreed 62.4% vs. purebred 62.6%), with no significant difference between these groups (*X*^2^ = 5.98, *p* = 0.308). Owners of designer crossbred puppies paid significantly more for their puppy compared to purebred puppy owners, with over a quarter of designer crossbred puppies (25.7%) costing £2000-2999 in comparison to only 15.1% of purebred puppies (*X*^2^ = 207.31, *p* < 0.001) (Fig. 4).

## Discussion

This study has for the first time characterised the purchase of designer crossbreeds in the UK, revealing important insights into the motivations and behaviours of the puppy buying public and shedding light on factors that may have contributed to the dramatic increase in demand for designer crossbred puppies. This new evidence base has highlighted several potential welfare issues associated with the rise in popularity of designer crossbreeds and the motivations behind it, most prominently, misconceptions of ‘breed’ characteristics driving their acquisition, and compromised ‘short-cut’ buying practices by prospective owners, which will now be considered in turn.

A common misconception of prospective dog owners is the belief that some designer crossbreeds, such as the Labradoodle, are hypoallergenic [8] and therefore have a reduced risk of eliciting an allergic reaction in humans. However, studies have found no evidence for differential shedding of hair and dog allergen CanF1 between designer and non-designer dogs [8, 9]. Indeed, the UK ‘Doodle Trust’ (a Poodle and Poodle cross rehoming charity) state that the supposition of ‘Doodles’ being allergy friendly and suitable for people with allergies is a ‘myth’ [23]. Despite this, owners of designer crosses in this study were more likely to be seeking a breed/crossbreed that they believed was hypoallergenic compared to purebred dog owners, a trait sought-after by almost half (47.1%) of this owner group. This is of concern as it could result in increased risk of future relinquishment if owners’ expectations are not met, and they or members of their household are allergic to their dog, given that allergies have been cited as one of the top three reasons for dog relinquishment in the USA, often (71.3%) within 1 year of ownership [24]. Countering this widespread misconception with educational messaging is of high importance, particularly via crossbreed-specific online resources, social media, and animal charity websites, as these information sources were commonly used in pre-purchase research by this owner group in this study. This corroborates recent findings that non-pedigree dog owners most commonly use general internet searches as an information source prior to acquiring their dog [25].

Further to the desire to acquire a hypoallergenic dog, the perception of designer crossbreeds being generally healthy appears an important driver of their popularity. Three in five owners of designer crossbred puppies sought out a breed/crossbreed based on the perception it was ‘generally healthy’, compared to just two in five purebred puppy owners. Some limited data support this assertion, for example, in a recent study of > 7000 Guide Dogs (of which almost half were known crossbreeds, most commonly Golden Retriever x Labrador Retrievers), crossbred dogs were more likely to have longer healthy lives than purebred dogs, albeit this varied markedly depending upon which health group was considered [26]. In addition, intentional crossbreeding of purebred dogs (sometimes followed by backcrossing the offspring of the cross with healthy individuals of one of the original breeds) has achieved improvements in health in some specific cases, e.g., crossing the Griffon Bruxellois with the Australian Terrier resulted in a reduced degree of Chiari malformation in offspring [27]. However, it could be argued that the perception of crossbred dogs being uniformly healthier than purebred dogs is likely erroneous. Recent disorder-specific studies have demonstrated that both the purebred Standard Poodle and its designer cross the Labradoodle are at increased odds of being diagnosed with Addison’s disease, a serious endocrine disorder in dogs [28], and that the prevalence of multifocal retinal dysplasia (MRD), a common hereditary condition in Labrador Retrievers, is higher in Labradoodles than Labrador Retrievers in the UK [17].

It is important to note that for disorders arising from single genes with a dominant mutation, crossbreeding where only one parental breed shows the mutated allele should result in clinically unaffected offspring in the first generation [29]. However, since many disorders with particular breed dispositions involve interacting roles from many genes and therefore represent polygenic disorders, it is more likely that designer crosses will show a disorder frequency close to the midpoint between the progenitor breeds [5]. In comparison to the more severely affected progenitor breeds, offspring could be considered to show a health advantage and may therefore be interpreted to be ‘generally healthier’. However, it would be equally valid to consider that these offspring were less healthy compared to the less affected progenitor breed. Indeed, it could even be considered that designer crosses were in effect introducing new disorders into offspring from the healthy progenitor breed.

Owners of designer crossbred puppies were less likely to prioritise seeking a breeder that would conduct the relevant health tests for their dog compared to purebred puppy owners. The British Veterinary Association (BVA) and Kennel Club state that designer crossbreeds such as the Goldendoodle and Labradoodle are amongst the top 10 breeds at risk of hip dysplasia [30] and thus highlighting to prospective owners and breeders the importance of health screening of breeding dogs, regardless of the pure or crossbred nature of their offspring, is key to the health of these populations. How owners’ perceptions of breed/crossbreed health develop and which information sources they are most influenced by is poorly understood but of increasing importance. Regarding dog health more generally, studies have found that over half (56.2%) of dog owners receive dog health information through dog-centred Facebook groups, with and around one in five (22.6%) considering Facebook a trustworthy source of health information [31]. Encouraging veterinary and animal welfare professionals to share educational messaging regarding breed/crossbreed health via social media may target a different demographic than traditional media; however, how such messaging is received should be monitored given online ‘activism’ in other animal sectors has been poorly received, e.g., meat consumers’ reactions to online farm animal welfare messaging [32].

Reliable prevalence data on the health problems facing designer crossbreeds is urgently needed to ensure such messaging is evidence-based. Data on the health of crossbred dogs is often hampered by incomplete recording of parentage in veterinary records, where the label of ‘crossbreed’ or a specific designer crossbreed is assigned. Data including the exact nature of the cross (e.g., F1, F2, backcross) is rarely recorded but vital to exploring crossbreed-specific health and broader hybrid vigour effects, given that different levels of hybrid vigour would be predicted in crosses involving different combinations of breeds that differ in their extent of between-breed genetic diversity [14]. While the first generation (F1 generation) of designer crosses between two distinctly differing parental purebred breeds may be significantly less inbred than either of their progenitor breeds [4, 33], subsequent generations resulting from crossing between the designer crosses themselves (F2 and subsequent crosses) are likely to return to higher inbreeding co-efficients and therefore to rapidly lose any health benefits that had originally resulted from hybrid vigour effects [14].

In addition to human and canine health-related traits, designer crossbreeds also appear to be purchased based on perceived positive behavioural traits, particularly those that fit with a family lifestyle, ease of training and suitability with children. Four in 10 (41.6%) designer crossbred puppies were purchased by households with children, compared to three in 10 (31.2%) purebred puppies. Correspondingly, over half (56.0%) of designer crossbreed owners sought a breed/crossbreed that they perceived to be good with children, at 1.46 increased odds of desiring this trait compared to purebred puppy owners. Being ‘safe’ with children is a behavioural trait that is valued in companion dogs internationally [34, 35]. However, the limited data that does exist on behavioural differences between designer crossbreeds and their progenitor breeds are limited to Standard Poodle crosses, and suggest that behavioural traits of the Labradoodle do not significantly differ from those of Labrador Retriever and Standard Poodle, and as predicted above for polygenic traits, Labradoodle behaviour tending to fall between the behavioural patterns of the two constituent progenitor breeds [3]. The same study also found that Goldendoodle (Golden Retriever x Standard Poodle) behaviour differed significantly in several ways from their progenitor breeds; however, the behaviours displayed by Goldendoodles were likely to be considered negatively by prospective owners, including increased dog-directed aggression and fear and stranger-directed aggression [3]. Breed-based behavioural assumptions pose multiple risks to canine and human welfare. They can impact the dog-owner relationship and increase relinquishment risk if expectations are not met [10]. Positive assumptions may also threaten public health risk (e.g., increase bite risks) if owners assume certain designer crossbreeds are ‘safe’ with children and do not provide appropriate training and supervision. Instilling realistic expectations for dog behaviour and training needs in prospective owners is of high priority for human and canine wellbeing.

The purchase of designer crossbreed puppies was associated with several pre-purchase and purchase behaviours that are likely to compromise canine welfare. Although almost nine in 10 designer crossbreed owners considered a breeder allowing them to see their puppies’ mother a sought-after characteristic (89.7%), only seven in 10 (73.1%) actually saw (what was at least claimed to be) their puppies mother upon collection of their puppy, indicating that some owners were willing to compromise on this vital step of the puppy-buying process. This was more prominent than in purebred puppy buyers, of which nine in 10 sought-after breeders that would let them ‘see mum’ (89.3%), and eight in 10 achieved this (79.8%). Puppies purchased without the buyer seeing their mother are more likely to display unwanted behavioural problems in the future [36], and thus this oversight during the purchase process could have long-term implications for dogs and their owners. Lucy’s Law was brought into force in England in April 2020, making it illegal to sell a puppy without its mother in the location it was born [37]. Whether and to what extent owners will continue to buy puppies without viewing their mother requires monitoring and potentially further behaviour change interventions if the law alone is unable to curb this behaviour. In further contravention of DEFRA’s advice as part of the ‘PetFishing’ campaign [38], owners of designer crossbred puppies were also more likely to place a deposit for their puppy before viewing them. This leaves designer crossbred puppy purchasers at a greater risk of ‘PetFishing’ [38], i.e., buying from a seller that pretends that the puppy they’re selling you comes from a reputable, responsibly bred source, but in reality, may have been bred or kept in poor conditions that are hidden from the buyer. Pre-viewing deposits also limit an owner’s freedom to change their mind later if they are dissatisfied with the conditions and circumstances under which their puppy was bred or sold, if they are unwilling to forfeit their deposit and/or are motivated to ‘save’ the puppy from the poor environment if encountered at collection. Therefore, the surge in public demand for designer crossbred puppies may have amplified the irresponsible breeding and illegal importation of puppies, which has been increasingly reported in the UK [39].

Owners of designer crossbred puppies were less likely to purchase their first-choice breed/crossbreed compared to purebred puppy owners, with participants citing an inability to find a seller that had their first-choice available at the time they wanted as the most common reason for this. This highlights the imbalance between the supply and public demand for puppies in 2019-2020, with purchases taking place in a ‘sellers’ market’, in which the demand for designer crossbred puppies outweighs their supply. This is exemplified in a report from online seller Pets4Homes, in which 283 buyers per Cavapoo puppy advert were reported in June 2021, followed by other designer crossbreeds such as the Goldendoodle (261 buyers/puppy), Labradoodle (228 buyers/puppy), Cockapoo (216 buyers/puppy) and Maltipoo (216 buyers/puppy) compared to other popular purebreds such as the French Bulldog (102 buyers/puppy) and Pug (102 buyers/puppy) [6]. This extreme competition may have resulted in puppy buyers making different choices to their original plans, which may be suboptimal, in order to fulfil their urgent desire to purchase a puppy. This was further reflected in designer crossbreed owners’ desired characteristics of a breeder, where a breeder having available puppies at the time they wanted of high priority for almost half of designer crossbreed owners (45.9%), significantly more so than purebred dog owners (37.2%). The emphasis upon convenience of the purchase was further exemplified in the increased likelihood of designer crossbreed owners seeking out a breeder that lived within the distance they were willing to travel (48.0%) compared to purebred dog owners (36.2%). Given that designer crossbreed owners were less likely to seek out a breeder they considered was trustworthy, with almost one quarter of owners not seeking this trait (23.4%), the risk of supporting irresponsible and potentially illegal practices in this population is high.

## Conclusion

The recent boom in sales of designer crossbreeds in the UK has been fuelled by a desire for hypoallergenic and generally healthy dogs that fit the lifestyles of households with children and owners with limited experience with dogs. Some of the traits sought-after in designer crossbreeds have minimal supporting evidence (e.g., suitability for people with allergies), and there is sometimes even evidence supporting the contrary (e.g., behaviour suited to homes with children). Such misconceptions are potentially dangerous for canine welfare, risking relinquishment or poor dog-owner relationships in the future if owner expectations are not met. Some of the purchasing practices fuelling this boom (e.g., lack of pre-purchase visits, pre-viewing deposits, high purchase prices and not seeing a puppy with their mother) support irresponsible and potentially illegal breeding and selling practices, which combined with assumptions of good ‘breed’ health but a lack of need for health testing in parents, may result in a high disease burden and poor future welfare for this growing population. Educational messaging, particularly via the internet and social media, are urgently needed to counter misconceptions driving the popularity of designer crossbreeds. Given the potential risks to the future welfare of designer crossbreeds, longitudinal cohort studies conducted as part of the Pandemic Puppies research programme will collect ongoing data on the designer cross population over their lives to explore future outcomes for this burgeoning population [40].

## Data Availability

The datasets used and/or analysed during the current study are available from the corresponding author on reasonable request.
